# Determining the Minimal Clinical Important Difference for Medication Quantification Scale III and Morphine Milligram Equivalents in Patients with Failed Back Surgery Syndrome

**DOI:** 10.3390/jcm9113747

**Published:** 2020-11-21

**Authors:** Lisa Goudman, Ann De Smedt, Patrice Forget, Maarten Moens

**Affiliations:** 1 Department of Neurosurgery, Universitair Ziekenhuis Brussel, Laarbeeklaan 101, 1090 Jette, Belgium; lisa.goudman@uzbrussel.be; 2 Center for Neurosciences (C4N), Vrije Universiteit Brussel, Laarbeeklaan 103, 1090 Jette, Belgium; ann.desmedt@uzbrussel.be; 3 STIMULUS Consortium (reSearch and TeachIng neuroModULation Uz bruSsel), Universitair Ziekenhuis Brussel, Laarbeeklaan 101, 1090 Brussels, Belgium; 4 Pain in Motion International Research Group (PAIN), Vrije Universiteit Brussel, Laarbeeklaan 103, 1090 Jette, Belgium; 5 Department of Physical Medicine and Rehabilitation, Universitair Ziekenhuis Brussel, Laarbeeklaan 101, 1090 Brussels, Belgium; 6 Institute of Applied Health Sciences, NHS Grampian, University of Aberdeen, Aberdeen AB25 2ZD, UK; patrice.forget@abdn.ac.uk; 7 Department of Radiology, Universitair Ziekenhuis Brussel, Laarbeeklaan 101, 1090 Jette, Belgium

**Keywords:** minimal clinically important difference, clinical importance, responsiveness

## Abstract

The Medication Quantification Scale III (MQS) is a tool to represent the negative impact of medication. A reduction in medication can serve as an indicator to evaluate treatment success. However, no cut-off value has yet been determined to evaluate whether a decrease in medication is clinically relevant. Therefore, the objective is to estimate the thresholds for the MQS and morphine milligram equivalents (MMEs) that best identify a clinically relevant important improvement for patients. Data from the Discover registry, in which patients with failed back surgery syndrome were treated with high-dose spinal cord stimulation, were used. Patient satisfaction was utilized to evaluate a clinically important outcome 12 months after stimulation. Anchor-based and distribution-based methods were applied to determine the minimal clinical important difference (MCID). Distribution-based methods revealed a value of 4.28 for the MQS and 33.61 for the MME as MCID. Anchor-based methods indicated a percentage change score of 41.2% for the MQS and 28.2% for the MME or an absolute change score of 4.72 for the MQS and 22.65 for the MME. For assessing a treatment outcome, we recommend using the percentage change score, which better reflects a clinically important outcome and is not severely influenced by high medication intake at baseline.

## 1. Introduction

Spinal cord stimulation (SCS) is a minimal-invasive treatment option that is recommended for patients with failed back surgery syndrome (FBSS) in the case that conservative treatment does not provide sufficient pain relief. This condition is characterised by lumbar or cervical pain of unknown origin, either persisting despite surgical intervention or appearing after surgical intervention for spinal (origin) pain originally in the same topographical distribution [1,2]. High-dose SCS (HD-SCS) is one of the new paradigms within the field of spinal cord stimulation that relies on delivering high energy at a subsensory threshold, with the main focus of increasing the impact of electrical charge delivery to the spinal cord [3]. 

Up till now, the success of SCS was mainly focused on obtaining sufficient pain relief. Nevertheless, reimbursement rules are also focusing on pain medication reductions, as, for example, at least 50% pain reduction and 50% reduction in pain medication use should be obtained in Belgium to consider a trial period successful. Additionally, the terms holistic responder and multidimensional responder index were recently launched, which comprise a combination of several factors among which pain relief, disability, and quality of life are outcome measures for SCS [4,5]. This clearly indicates that there is an ongoing reformulation of the definition of success in the field of neuromodulation towards a more holistic perspective [6,7]. However, when constructing this definition, clinicians are confronted with a wide variety of available cut-off values that could be applied. Moreover, challenges exist in determining the clinical significance of a change in outcome measurements from a patient’s perspective [8]. One possible solution to handle this issue is by relying on minimal clinical important difference (MCID) values. An MCID can be considered as the smallest change or difference in an outcome measure that is perceived as beneficial and would lead to a change in the patient’s medical management, assuming an absence of excessive side effects and costs [9].

Although the use of opioids is controversial and currently only recommended for short time periods, opioids are still broadly used in the treatment of patients with FBSS [10]. A recent systematic review indicated that SCS is significantly associated with decreased medication use and opioid consumption compared to the best medical treatment [11]. As such, reducing medication use is factually a component of treatment success and should be consistently quantified. The Medication Quantification Scale III (MQS) is a tool specifically designed to quantify pain medication regimens by providing a numerical output that represents the negative impact of each medication in a wide variety of pain conditions [12,13]. Nevertheless, no clinical important cut-off values for the MQS or morphine milligram equivalents (MMEs) have been proposed yet. Therefore, the objective of the present study is to estimate the thresholds for the MQS and MME that best identify FBSS patients who perceived a clinically important outcome following HD-SCS stimulation.

## 2. Experimental Section

### 2.1. Data 

In this study, data from a prospective multicenter registry-based cohort study towards the effectiveness of HD-SCS were used. The protocol of this real-world registry (called Discover) was registered on clinicaltrials.gov (NCT02787265) on 1 July 2016 and previously published [14]. In this registry, 272 FBSS patients were recruited between October 2016 and November 2019 in 15 Belgian neuromodulation centres and 1 centre in France. FBSS patients with a numerical rating scale (NRS) score ≥5/10 for leg and/or back pain for at least 6 months and who were suitable for HD-SCS were eligible to participate. FBSS patients with a numerical rating scale (NRS) score >3/10 for leg and/or back pain who were treated with standard SCS and dissatisfied with standard SCS as treatment were also eligible to participate. All patients received high-dose spinal cord stimulation (HD-SCS) as treatment. Three follow-up visits took place in the following year, namely, after one month, three months, and twelve months of HD-SCS.

The study was conducted according to the revised Declaration of Helsinki (1998). The study protocol was approved by the ethics committee of Universitair Ziekenhuis Brussel (B.U.N. 143201629180) and the ethics committees of each participating centre. All patients provided written informed consent before enrolment in this study.

### 2.2. Patient-Reported Outcome Measures

Patients were asked to provide the details of all pain medication, with corresponding frequency and amount per day, at each visit. The MQS is designed to quantify pain medication regimens in a wide variety of pain conditions [12]. It provides a numerical output that represents the negative impact of each medication [13]. For each medication, an MQS score is calculated by multiplying a detriment weight for a given pharmacologic class with a score for dosage [15]. Medication is subdivided into five classes: nonsteroidal anti-inflammatory drugs (NSAIDs), muscle relaxants, neuropathic pain medications (antidepressants and anticonvulsants), benzodiazepines, and opioids. All calculated values are summed to obtain a total MQS score. For opioids, MMEs were calculated as well. For MMEs, the total quantity is multiplied by the strength per unit and then multiplied by the opioid-specific morphine conversion factor. Morphine conversion factors from the CDC were used [16].

Patient satisfaction with HD-SCS was measured with a 5-point Likert scale (not at all satisfied, slightly satisfied, neutral, very satisfied, and extremely satisfied) at the follow-up visits. Patients who rated themselves as extremely satisfied or very satisfied at a 12-month follow-up were considered to have gained a clinically important outcome following SCS implantation (better), whereas other patients were considered to have not benefited from their treatment (worse/neutral) [17].

### 2.3. Statistical Analysis

All analyses were performed in R Studio version 1.2.5019 (R version 3.6, Boston, MA, USA,) and SAS version 9.4 (100 SAS Campus Drive Cary, NC, USA). Both for MME and MQS scores, three different calculations were evaluated: (1) the raw scores at baseline and 12-month follow-up, (2) the absolute change score (i.e., the absolute change from baseline minus follow-up, and (3) the percentage change score (i.e., the relative change score calculated as ((baseline score—follow-up score)/baseline score) × 100). In total, five different approaches were used to determine the MCID of which two are distribution-based methods (standard deviation (SD) and effect sizes) and three are anchor-based methods (within-patients change score, between-patients change score, and sensitivity/specificity). Distribution-based methods rely on statistical parameters reflecting the statistical variation and measurement accuracy of the outcome [18]. Anchor-based methods use an explicit scale which is referred to as an “anchor”, meaning that after treatment, this scale serves as an external standard against which changes in the outcome score can be compared [19]. The first method to define the MCID is by calculating the standard deviation of the baseline score. The calculation of the MCID = 0.5 × SD, where SD represents the SD of the baseline score of the patient population being studied [20]. This method is based on a systematic review of studies that have calculated the MCID with different measurement instruments, whereby it was revealed that the MCID converged roughly to half the SD of the baseline score, regardless of the methodology that was used to calculate the MCID [20]. The second method is based on effect sizes (i.e., a standardised measure of change obtained by dividing the absolute difference in scores from baseline to follow-up by the SD of the baseline scores). Regarding MCID, the change in scores corresponding to the small effect size is considered the MCID [21]. Thus, multiplying the SD of the baseline scores by 0.2 (denoting a small effect size) provides the MCID (i.e., MCID = 0.2 × SD) [22]. The third method to determine the MCID is an anchor-based method, whereby the absolute and relative changes in mean scores for patients categorised as “better” are used as MCID values [18] (within-patient change score). In the fourth method, the MCID is defined as the difference in the absolute change score of the patients classified as “better” and the group classified as “worse/neutral” [21] (between-patient change score). The fifth method consists of selecting the value that allows for the best discrimination between “better” and “worse/neutral” (i.e., the score that produces the best results concerning sensitivity and specificity) as MCID. The value with equal sensitivity and specificity is chosen as the MCID value [21].

## 3. Results

### 3.1. Patient Characteristics

Baseline data on medication use were available for 259 patients (43.6% males, 56.4% females). Patients had a mean age of 54.7 years (SD: 11.8 years). At 12-month follow-up, the data of 130 patients were available, of which 0.8% of patients were not at all satisfied with HD-SCS, 18.5% were slightly satisfied, 8.5% were neutral, 31.5% were very satisfied, and 40.8% were extremely satisfied. Mean MQS score at baseline was 10.99 (95% CI from 9.94 to 12.03) and 6.57 (95% CI from 5.39 to 7.75) at 12-month follow-up. The mean MME score at baseline was 43.35 (95% CI from 35.12 to 51.58) and 22.50 (95% CI from 15.65 to 29.35) at 12-month follow-up.

### 3.2. Distribution-Based Methods

In the first method, the MCID is equal to 0.5 times the standard deviation of the baseline score. This resulted in a value of 4.28 for the MQS and 33.61 for the MME. In the second method, which is based on a small effect size, the proposed MCID value was calculated by multiplying the standard deviation of the baseline score by 0.2. This resulted in an MCID value of 1.71 for the MQS and 13.45 for the MME.

### 3.3. Anchor-Based Methods

**Figure 1:**
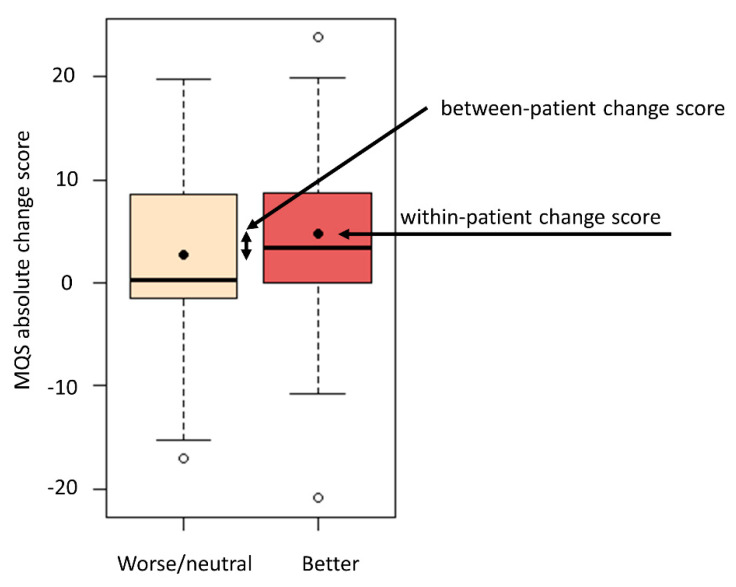
Boxplot of Medication Quantification Scale III (MQS) absolute change score between baseline and 12-month follow-up. On the *y*-axis, baseline MQS score minus follow-up MQS score is presented. On the x-axis, “better” and “worse/neutral” levels are presented based on patient satisfaction scores. Within the boxex, the median is allocated with a black line and the mean with a black circle. The white circles on the plot are presenting more extreme data observations. Within-patient change score is the mean absolute change score for the “better” group. The difference in the absolute change score of the patients classified as “better” and the group classified as “worse/neutral” is the between-patient change score.

Within the third method, absolute and percentage change scores for patients within the “better” group are considered as MCID values. This resulted in an absolute MCID value of 4.72 for the MQS (Figure 1) and 22.65 for the MME.

**Figure 2:**
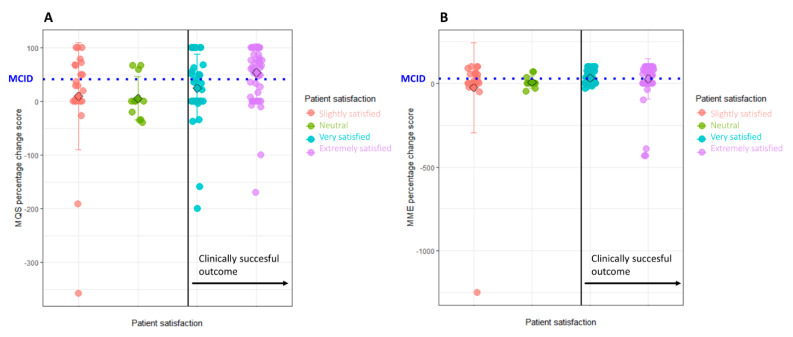
Scatterplot of MQS (**A**} and morphine milligram equivalent (MME) (**B**) percentage change score between baseline and 12-month follow-up, separated according to patient satisfaction. The colours represent the different levels of patient satisfaction. Levels on the right side of the solid black line are considered clinically successful and categorised as “better”. On the *y*-axis, percentage scores between baseline and 12-month follow-up are presented, which were calculated as follows: ((baseline score − follow-up score)/baseline score) × 100). Positive scores indicate an improvement at follow-up compared to baseline, while negative percentages indicate a worse outcome at follow-up compared to baseline. MCID values were calculated and are presented on the plot by the dashed blue lines. For MQS, the MCID is located at 41.18% and for the MME at 28.20%. Abbreviations. MME: morphine milligram equivalents; MQS: Medication Quantification Scale III.

When calculating with percentage changes, values of 41.2% for the MQS and 28.2% for the MME were proposed (Figure 2).

In the fourth method, the MCID is defined as the difference in the absolute change score of the patients classified as “better” and the group classified as “worse/neutral” (between-patient change score), which resulted in an MCID value of 2.03 for the MQS (Figure 1) and 15.18 for the MME. The fifth method consists of selecting the value that allows for the best discrimination between “better” and “worse/neutral” as MCID, which resulted in a proposed MCID value of 1.4 for absolute changes and 30% as percentage change score for the MQS. For the MME, 8 was proposed as the MCID value for absolute changes and 17% for percentage change scores. Table 1 presents an overview of all proposed MCID values.

## 4. Discussion

In this study, we determined the MCID for medication use in patients with FBSS who are treated with HD-SCS. As already indicated by several other researchers, this study confirms that when applying several methods to determine MCID, all methods will provide different cut-off values [17,23]. The variety in obtained values in anchor-based methods is probably due to the criterion scale and the arbitrary selection of scale levels (i.e., the grouping of the levels) [21]. Distribution-based methods, on the other hand, depend on the measure of statistical variability that is used, thereby neglecting clinical importance and consecutively ignoring the purpose of the MCID, which is to denote a clinically important difference instead of a statistically significant difference [21]. Therefore, clinicians and researchers should carefully evaluate which MCID value is the most suitable in their specific context. In longitudinal designs, anchor-based methods might better reflect clinical importance, while distribution-based methods might be better suited for cross-sectional designs. 

Medication use was globally evaluated with the MQS, combined with a specific focus on opioids through the MME. The opioid crisis is still a major global public health concern [24], with an increase in the number of opioid prescriptions during the last decade [25]. Due to this epidemic, many researchers only focus on opioids instead of taking neuropathic pain medication, muscle relaxants, and other categories into account. Moreover, for chronic noncancer pain, recommendations and guidelines strive towards avoiding doses above 90 MME [26,27,28], wherefore a clinically important change in MME might be very relevant for clinicians that mainly aim to focus on opioid reduction. 

One of the methods to determine the MCID relies on sensitivity and specificity. Specifically, in the context of MCID, sensitivity is the proportion of patients who report an improvement in patient satisfaction and whose medication scores are above the threshold MCID value. Specificity is the proportion of patients who do not report an improvement in patient satisfaction and whose medication scores are below the threshold MCID value [21]. A sensitivity of 1 indicates that all true positives are identified, whereas a specificity of 1 indicates that all true negatives are identified. For both opioid use and global medication use, sensitivity and specificity were rather low, wherefore this method might not be the most suitable in this case. 

In determining the MCID, one needs to take into account that there is an association between baseline scores and change scores, such that patients with greater baseline levels of disability reveal a greater improvement after follow-up [29,30]. Similar results were found for pain intensity, whereby patients with a high baseline level of pain on the NRS, who experienced either a slight improvement or a higher level of response, had absolute raw and percentage changes greater than patients with lower baseline scores [31]. Previously, it was already stated that the use of the MCID value alone might be limited when determining treatment effects, due to baseline dependency of the sample [32]. One possible solution to manage this association is by using the percentage change instead of the absolute change scores to determine an MCID value. As such, we propose to work with percentage change scores, whereby an MCID value of 41.2% should be obtained for the MQS and 28.2% for the MME. 

The MCID in this study is based on a cohort study of patients with FBSS. A larger sample size would have allowed for a better generalisation of the results and an improvement in precision. Additionally, the design of the Discover registry did not allow us to use methods that rely on the standard error of measurement, which is also considered a proxy for the MCID [33].

## 5. Conclusions

For estimating “a successful outcome” of HD-SCS in patients with FBSS, according to a clinically relevant point of view instead of a statistical point of view, a percentage change of 41.2% should be reached for the MQS and 28.2% for the MME.

## Figures and Tables

**Table 1:** Distribution- and anchor-based proposed MCID values for the MQS and MME in patients with FBSS after treatment with high-dose spinal cord stimulation (HD-SCS).

Approach	Method	Calculation	MQS	MME
Distribution-based methods	Standard deviation	MCID = 0.5 × SDbaseline	4.28	33.61
	Effect size	MCID ≥ 0.2 × SDbaseline	1.71	13.45
Anchor-based methods	Within-patient change score	Absolute change score for “better” group	4.72	22.65
		Percentage change score for “better” group	41.2%	28.2%
	Between-patient change score	Difference in change score between “better” and “worse/neutral” group	2.03	15.18
	Sensitivity/Specificity	Absolute change score	1.4 (52.9% sensitivity and 66.7% specificity)	8 (61.8% sensitivity and 53.8% specificity)
		Percentage change score	30% (61.8% sensitivity and 62.4% specificity)	17% (61.8% sensitivity and 57.0% specificity)

Abbreviations. FBSS: Failed Back Surgery Syndrome; MCID: minimal clinical important difference; MME: morphine milligram equivalent; MQS: Medication Quantification Scale III; SD: standard deviation.
